# Plasma proteomic profiling of hospitalized patients co-infected with HIV and SARS-CoV-2

**DOI:** 10.3389/fimmu.2025.1601672

**Published:** 2025-06-11

**Authors:** Xinyu Zhang, Xuan Yan, Jinfeng Sun, Yueming Shao, Wei Song, Tangkai Qi, Zhenyan Wang, Yang Tang, Jianjun Sun, Jingna Xun, Zichen Song, Shuibao Xu, Junyang Yang, Jiangrong Wang, Jun Chen, Renfang Zhang, Li Liu, Yinzhong Shen

**Affiliations:** ^1^ Department of Infection and Immunity, Shanghai Public Health Clinical Center, Fudan University, Shanghai, China; ^2^ Shanghai Institute of Infectious Disease and Biosecurity, Fudan University, Shanghai, China

**Keywords:** HIV, COVID-19, SARS- CoV- 2, coinfection, proteomic

## Abstract

**Background:**

The interaction between human immunodeficiency virus (HIV) and severe acute respiratory syndrome coronavirus 2 (SARS-CoV-2) infections presents a critical challenge to immunopathogenesis. While HIV infection induces progressive CD4^+^ T cell depletion and chronic immune dysfunction, SARS-CoV-2 triggers complex host responses, ranging from localized antiviral defense to systemic hyperinflammation. We aimed to illustrate the plasma proteomic profiles of hospitalized patients coinfected with HIV and SARS-CoV-2.

**Methods:**

Liquid chromatography-tandem mass spectrometry was used to analyze the plasma protein profiles in three matched groups: (1) seven hospitalized patients with HIV and SARS-CoV-2 coinfection, (2) seven people living with HIV (PLWH) who tested negative for SARS-CoV-2, and (3) seven healthy controls. Gene Ontology and Kyoto Encyclopedia of Genes and Genomes enrichment analyses were performed on the differentially expressed proteins (DEPs).

**Results:**

We quantified 5,373 proteins across 21 samples and identified significant alterations in multiple proteins in people living with HIV (PLWH) with COVID-19 compared to both PLWH and healthy controls. These DEPs were associated with inflammatory responses, immune cell migration, degranulation, and, notably, the complement and coagulation cascades. In addition, we identified DEPs associated with SARS-CoV-2 infection, including viral receptors, proteases, transcription factors, and kinases.

**Conclusions:**

The proteomic profile highlighted the disruption caused by COVID-19 in immunomodulation, thrombosis, and viral entry pathways in PLWH. Further validation of these signatures could improve risk stratification and tailored interventions for this vulnerable patient cohort.

## 1 Introduction

As the coronavirus disease 2019 (COVID-19) pandemic continues to affect populations worldwide, considerable efforts have been focused on understanding the humoral and cellular immune responses and mechanisms of protection against severe acute respiratory syndrome coronavirus 2 (SARS-CoV-2) infection. The phenomenon of cytokine storm is considered a potential cause of the hyperactive immune response observed in SARS-CoV-2 infection and is closely related to the severity of COVID-19 (1–3). The production of proinflammatory cytokines attracts immune cells to the site of infection, leading to tissue damage that may result in pneumonia, lung injury, and multiple organ failure, which are common complications in critically ill and deceased patients with COVID-19 (4, 5). The immune response to SARS-CoV-2 infection varies significantly among individuals (6, 7). Research indicates that among hospitalized patients with COVID-19, people living with human immunodeficiency virus (PLWH) aged <65 years with a CD4 cell count <350 cells/mm^3^ have a higher risk of in-hospital mortality than the general population of a similar age. Furthermore, a clear dose-response relationship is associated with the CD4 count, with the risk being even higher in those with a CD4 count <200 cells/mm^3^ (8–10). Despite an increasingly consolidated body of evidence on COVID-19 in the general population, the interaction between SARS-CoV-2 and human immunodeficiency virus (HIV) infections remains unclear.

HIV infection is characterized by CD4^+^ T cell depletion, CD8^+^ T cell expansion, and chronic immune activation, leading to immune dysfunction (11). The effects of COVID-19 on the immune and inflammatory systems are of particular concern in PLWH, given their immunocompromised state and chronic inflammation status. SARS-CoV-2 can productively infect CD4^+^ T cells by binding to cell entry receptors other than angiotensin-converting enzyme 2 (ACE2) and inducing apoptosis. This suggests that HIV and SARS-CoV-2 may converge in their effects on the immune system (12). Information on the changes in soluble proteomic markers following SARS-CoV-2 infection can help to better understand the immune responses in PLWH with COVID-19. In this study, we profiled host responses to SARS-CoV-2 infection in humans by performing quantitative proteomics on plasma samples from a cohort of PLWH with COVID-19. Our study identified numerous changes in host proteins associated with COVID-19, particularly those related to inflammation and coagulation.

## 2 Materials and methods

### 2.1 Participants

In this study, we enrolled seven SARS-CoV-2 PCR-positive individuals from hospitalized people living with HIV (PLWH with COVID-19), along with seven SARS-CoV-2-negative hospitalized PLWH (referred to as PLWH) and seven healthy controls (HCs) as comparison groups. The two PLWH groups were matched for age, gender, and HIV viral load (**Table 1**).

**Table 1:** Participant characteristics.

Parameters	PLWH with COVID-19 (n=7)	PLWH (n=7)	HC(n=7)
Number of males	7	6	4
Number of females	0	1	3
Age (years)	55.43 ± 22.98	62.43 ± 12.61	27.29 ± 3.35
On ART	4	4	/
ART-naïve	3	3	/
HIV RNA (average, 106copies/mL)	0.67±0.82	0.68±1.36	/

ART, antiretroviral therapy; Values are mean ± SD.

This study was approved by the Ethics Committee of the Shanghai Public Health Clinical Center (Ethics Approval Number: 2023-S082-01). All participants provided written informed consent.

### 2.2 Plasma sample preparation and protein digestion

Blood was collected in 6 mL EDTA tubes (BD Vacutainer). The tubes were gently mixed by inverting 8–10 times, followed by centrifugation at 2000× rpm at 20°C for 20 min. Plasma samples were stored at –80°C until mass spectrometry analysis.

DL-Dithiothreitol (with the final concentration of 40 mM) was added to each sample respectively and mixed at 600 rpm for 1.5 h (37°C). After the samples cooled to room temperature, iodoacetamide was added with the final concentration of 20 mM into the mixture to block reduced cysteine residues and the samples were incubated for 30min in darkness. Next, trypsin was added to the samples (the trypsin: protein ratio was 1:50) and incubated overnight at 37°C. The peptides of each sample were desalted on C18 Cartridges (Empore™ SPE Cartridges MCX, 30UM, waters), concentrated by vacuum centrifugation and reconstituted in 40 µl of 0.1% formic acid. The peptide content was estimated by UV light spectral density at 280 nm. For data independent acquisition (DIA) experiments, indexed retention time calibration peptides were spiked into the sample.

### 2.3 Mass spectrometry analysis

Peptides were analyzed by OrbitrapTM AstralTM mass spectrometer (Thermo Scientific) connected to a Vanquish Neo system liquid chromatography (Thermo Scientific) in DIA mode. Precursor ions were scanned at a mass range of 380-980m/z, MS1 resolution was 240000 at 200 m/, normalized automated gain control (AGC )Target: 500%, maximum ion injection time( IT): 5ms. 299 windows were set for DIA mode in MS2 scanning, isolation window: 2m/z, Higher-energy collisional dissociation collision energy: 25ev, normalized AGC target: 500%, maximum IT: 3ms.

DIA data was analyzed with DIA-NN 1.8.1. Main software parameters were set as follows: enzyme is trypsin, max missed cleavages are 1, fixed modification is carbamidomethyl(C), dynamic modification is oxidation (M) and acetyl (Protein N-term). All reported data were based on 99% confidence for protein identification as determined by false discovery rate (FDR) ≤ 1%.

### 2.3 Pathway analysis

Gene Ontology (GO) annotation and pathway mapping were performed using Blast2GO (v2.8.0+). Proteins were matched against the Kyoto Encyclopedia of Genes and Genomes (KEGG) database (https://www.genome.jp/kegg/) to obtain KEGG orthology identifiers and pathway annotations. Significantly enriched GO terms and KEGG pathway were identified by Fisher’s exact test, and multiple testing correction was applied using the Benjamini-Hochberg. Only functional categories and pathways with *p*-values below the threshold of 0.05 were considered statistically significant. The Rich Factor is defined as the ratio of the number of differentially expressed proteins annotated to a specific GO term or KEGG pathway to the total number of identified proteins annotated to that GO term or KEGG pathway.

### 2.4 The transcription factors and kinases analysis

Animal Transcription Factor Database (http://bioinfo.life.hust.edu.cn/AnimalTFDB#!/download) was used to annotate transcription factors and their transcription factor family (13). PhosphoSitePlus (PSP, http://www.phosphosite.org) and Phospho.ELM (http://phospho.elm.eu.org) database were used to annotate kinases.

### 2.5 Statistical analysis

Fold-change (FC) values were calculated as the ratio of mean protein expression between PLWH with COVID-19 versus PLWH or HCs. Two-sided *t*-test was performed for each pair of groups to be compared, and corrected for FDR using the Benjamini and Hochberg method. One- way-ANOVA and Tukey’s HSD was performed for three groups to be compared. Statistical analysis was performed using R version 3.6.1 and GraphPad Prism 8.0.

## 3 Results

### 3.1 Systemic dysregulation of plasma protein in PLWH with COVID-19

**Figure 1:**
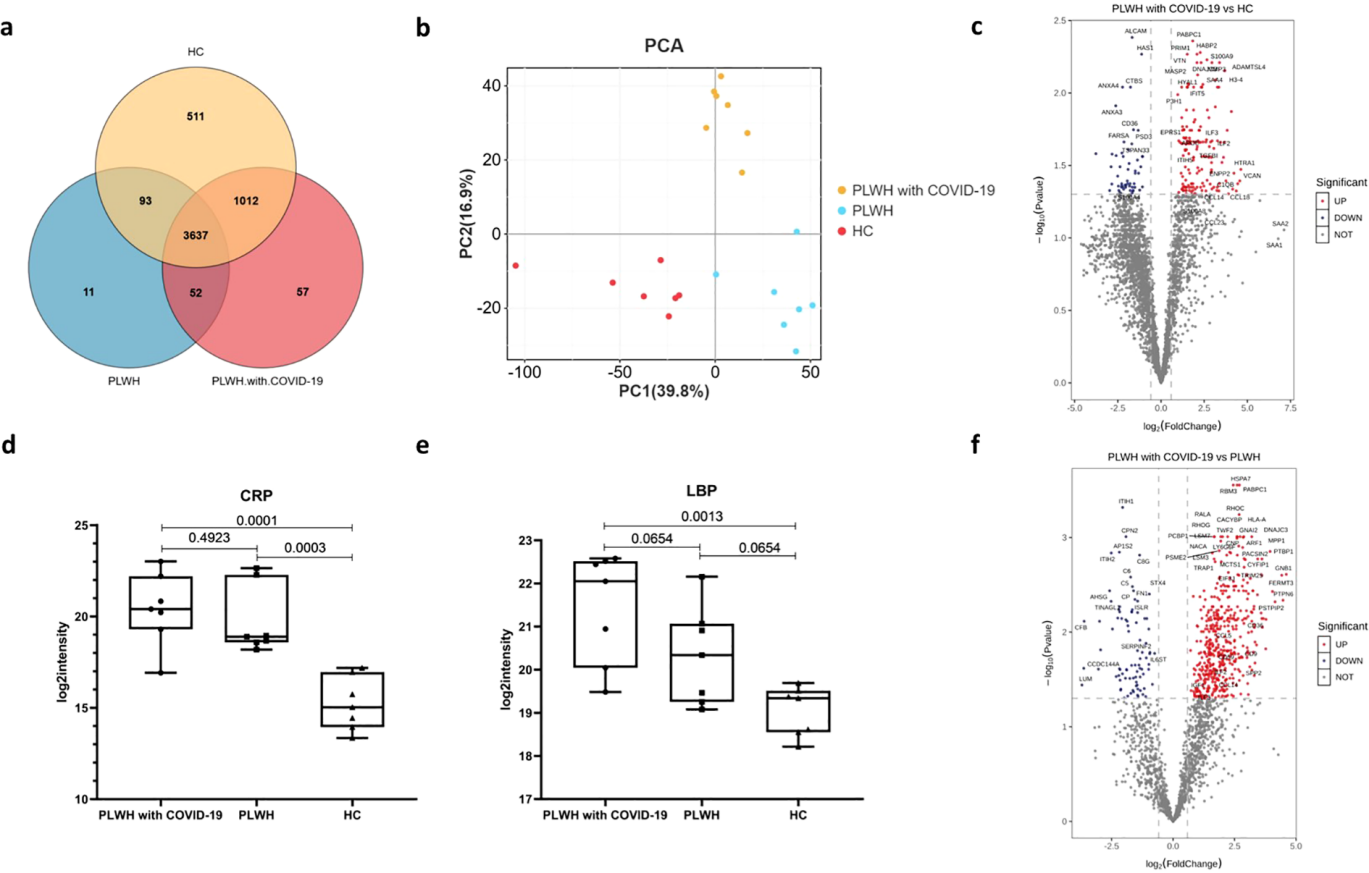
Visualization of the number of common differentially expressed proteins in the PLWH with COVID-19, PLWH and HCs. **(a)** Venn diagram showing the overlap of expressed proteins among the PLWH with COVID-19, PLWH and HCs. **(b)** Principal component analysis illustrating the variation in the expressed proteins between the three groups. **(c)** Volcano plot displaying the significantly upregulated (red dots, FC>1.5, two-sided *t* test, *p <*0.05, adjusted by FDR) and downregulated (blue dots, FC< 0.67, two-sided *t* test, *p <*0.05, adjusted by FDR) proteins between the PLWH with COVID-19 and HCs. d-e. Box plots depicting the expression levels of CRP **(d)** and LBP **(e)** across the PLWH with COVID-19, PLWH and HCs. One-way ANOVA and Tukey’s HSD used for statistical analysis. **(f)** Volcano plot highlighting the significantly upregulated and downregulated proteins between the PLWH with COVID-19 and HIV, two-sided *t* test, *p <*0.05, adjusted by FDR.

We quantified 5373 proteins in 21 samples using plasma proteomics. A Venn diagram revealed that 3689 proteins were common between the PLWH with COVID-19 and PLWH datasets (**Figure 1a**). Principal component analysis revealed that the three groups of PLWH with COVID-19, PLWH, and healthy controls (HC) were independently distributed, indicating significant differences in protein expression profiles between the two PLWH groups and the HC group (**Figure 1b**). This was further supported by the differential expression analysis of proteins comparing PLWH with COVID-19 with PLWH and HCs.

Compared to the HC group, 209 proteins exhibited altered plasma levels in PLWH with COVID-19, with 155 proteins significantly upregulated (fold change [FC] > 1.5, two-sided *t*-test, p < 0.05, adjusted by false discovery rate [FDR]; **Figure 1c**) and 54 significantly downregulated (FC < 0.67, two-sided *t*-test, p < 0.05, adjusted by FDR; **Figure 1c**). Among all differentially expressed proteins (DEPs), PABPC1, PRIM1, vitronectin (VTN), HABP2, and S100 calcium-binding protein A9 (S100A9) showed the most significant upregulation, whereas ALCAM, HAS1, CTBS, ANXA3, and ANXA4 showed the most significant downregulation. S100A9 is an acute-phase protein whose plasma levels change in response to inflammation, infection, and tissue injury (14). Serum amyloid A (SAA) levels increase during the acute phase of inflammatory diseases (15). Notably, SAA4 levels were significantly elevated compared with those in HCs (**Figure 1c**). We also observed that plasma from PLWH with COVID-19 had the greatest multiplicative increase in SAA2 (FC = 139.28) and SAA1 (FC = 110.40); however, this difference was not statistically significant after FDR correction. Other acute-phase proteins, including C-reactive protein (CRP; **Figure 1d**) and lipopolysaccharide-binding protein (LBP; **Figure 1e**), were also significantly elevated in PLWH with COVID-19 compared with HCs. Altered plasma protein levels likely reflect both hyperinflammatory and reparative states in PLWH with COVID-19.

Compared to PLWH, 568 proteins had altered plasma protein levels in PLWH with COVID-19, of which 482 were significantly upregulated and 86 were significantly downregulated (**Figure 1d**). Among all DEPs, PABPC1, HSPA7, RBM3, RHOC, and MPP1 were significantly upregulated, whereas ITIH1, CPN2, AP1S2, ITIH2, and C8G were significantly downregulated (**Figure 1f**). GNB1 showed the largest increase in the plasma of PLWH with COVID-19, followed by PTPN6, FERMT3, and PSTPIP2. LUM, CCDC144A, CFB, and KRT13 showed the largest decrease. However, changes in several acute-phase proteins, such as CRP and LBP, were not significant between the two groups of PLWH (**Figures 1d, e**).

### 3.2 Complement cascade activation and coagulation dysregulation in patients with HIV and SARS-Cov-2

**Figure 2:**
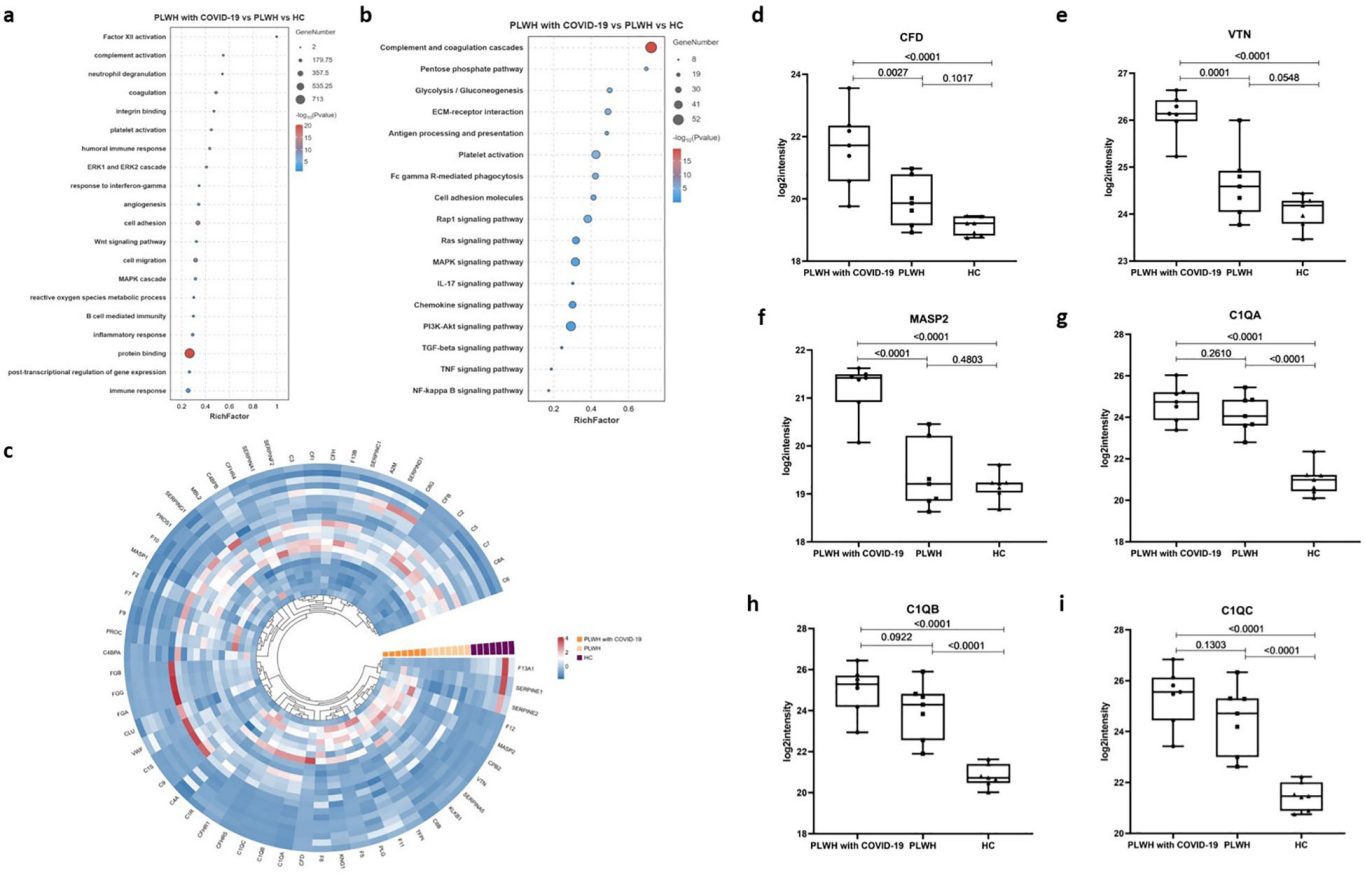
GO and KEGG functional enrichment analysis of differentially altered proteins between the three groups. **(a)** Bubble plots showing GO enrichment analysis for the PLWH with COVID-19, PLWH, and HCs. **(b)** Bubble plots showing KEGG enrichment analysis for the PLWH with COVID-19, PLWH, and HC. Fisher’s Exact Test (-log10) was used, the color gradient represents the size of the Rich Factor (Rich Factor ≤ 1), and the size of the bubbles indicates the number of DEPs under each GO function (or KEGG pathway) classification. **(c)** Circular heatmap illustrating dysregulated proteins related to the complement and coagulation cascade reaction in the PLWH with COVID-19, PLWH, and HCs. d-h. Box plots showing the expression levels of CFD **(d)**, VTN **(e)**, MASP2 **(f)**, C1QA **(g)**, C1QB **(h)**, and C1QC **(i)** in the PLWH with COVID-19, PLWH, and HCs. One-way ANOVA and Tukey’s HSD used for statistical analysis.

To characterize the plasma proteome in relation to previous reports and the known properties of COVID-19 and HIV, we performed pathway enrichment analysis using Gene Ontology (GO) terms and Kyoto Encyclopedia of Genes and Genomes (KEGG) pathway databases (16–18). GO enrichment analysis is commonly used to show interactions between genes and terms, whereas KEGG enrichment analysis illustrates the relationship between genes and patterns of function (19). The GO terms and KEGG pathways associated with DEPs were highly enriched in processes involved in inflammation, immune cell migration and degranulation, the complement system, coagulation cascades, and energy metabolism (**Figures 2a, b**; **Supplementary Data Sheet**). These biological processes are consistent with those of previous reports showing that acute inflammation and excessive immune cell infiltration are associated with COVID-19 severity (20, 21). Meanwhile, the activation of numerous defense responses, including pathways typical of adaptive immunity (such as B cell receptor signaling) and others specific to humoral innate responses (such as Fc gamma R-mediated phagocytosis), was observed in PLWH with COVID-19 and PLWH (**Figures 2a, b**). We also observed higher enrichment of DEPs in the complement and coagulation cascade pathways (**Figures 2a, b**). Several proteins related to the complement cascade have been shown to be dysregulated in PLWH with COVID-19 (**Figure 2c**); for example, complement factor D (CFD), mannan-binding lectin serine protease 2, and VTN levels were significantly elevated compared to those in PLWH and HCs (**Figures 2d–f**). The levels of proteins that initiate the cascade, such as complement C1q subcomponent subunits A, B, and C (C1QA, C1QB, and C1QC), were higher than those in the HC group but showed no significant differences compared to PLWH (**Figures 2g–i**).

**Figure 3:**
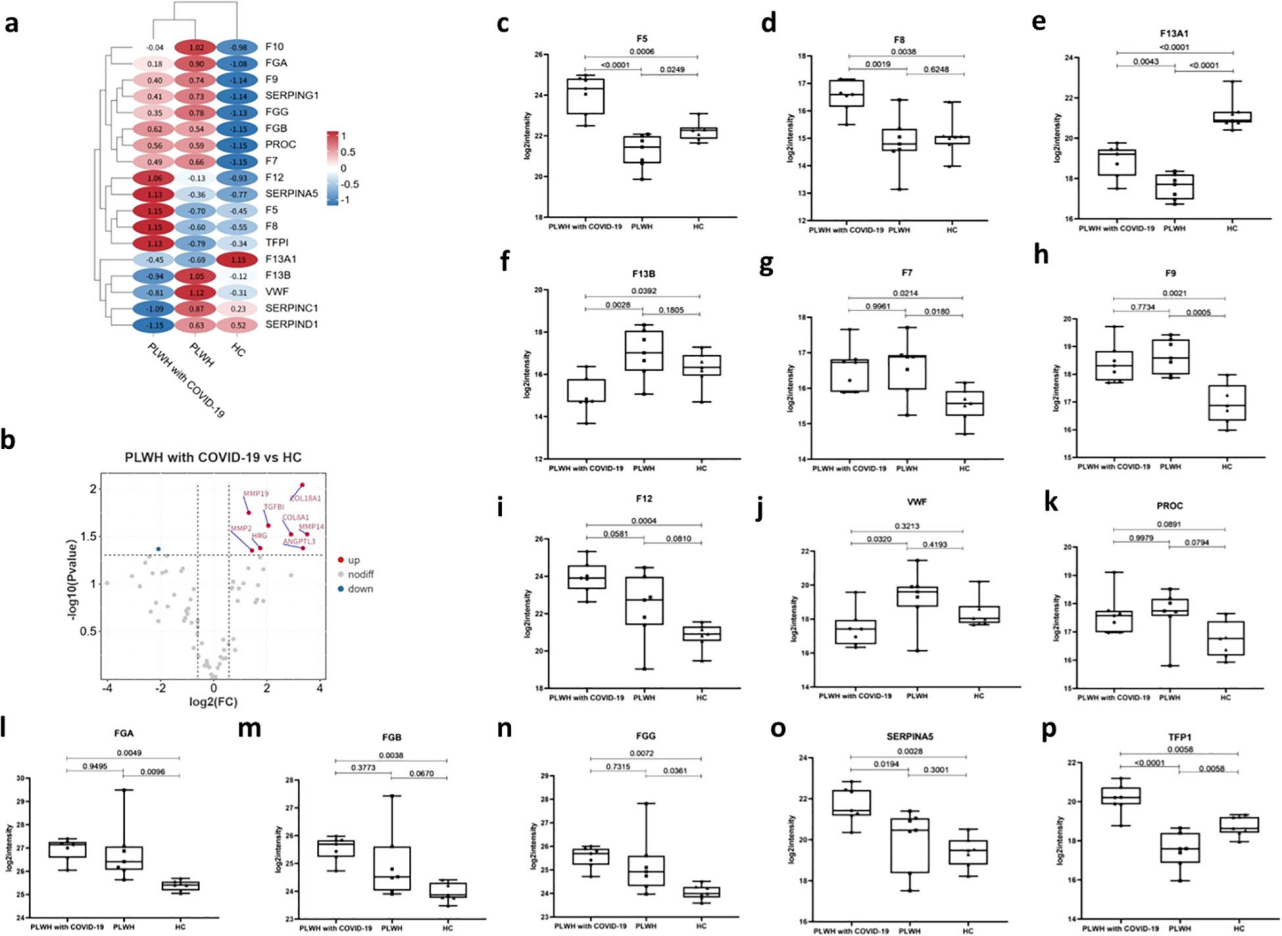
Dysregulation of coagulation, angiogenesis, and fibrosis in PLWH with COVID-19. **(a)** Heatmap showing dysregulated proteins related to the coagulation cascade in PLWH with COVID-19, PLWH, and HCs. Heatmap horizontal coordinates are sample groupings and each circle represents protein expression after Z-score transformation. **(b)** Volcano plot displaying significantly upregulated (red dots, FC > 1.5, two-sided *t* test, *p <*0.05, adjusted by FDR) and downregulated (blue dots, FC < 0.67, two-sided *t* test, *p <*0.05, adjusted by FDR) angiogenesis-related proteins between the PLWH with COVID-19 and HCs. c-p. Box plots showing the expression levels of F5 **(c)**, F8 **(d)**, F13A1 **(e)**, F13B **(f)**, F7 **(g)**, F9 **(h)**, F12 **(i)**, VWF **(j)**, PROC **(k)**, FGA **(l)**, FGB **(m)**, FGG **(n)**, SERPINA5 **(o)** and TFPI **(p)**, in the PLWH with COVID-19, PLWH, and HCs.One-way ANOVA and Tukey’s HSD used for statistical analysis.

The initial coagulopathy of COVID-19 is characterized by increased D-dimer and fibrinogen or fibrin degradation products, as well as abnormalities in prothrombin time, acute partial thromboplastin time, and platelet counts (22). The processes of systemic inflammation and hypercoagulation are interdependent, and the coagulation cascade can be activated by the consequent increase in proinflammatory cytokines (23). Our data revealed multiple dysregulated proteins involved in coagulation, anticoagulation, and the fibrinolytic system, which may have contributed to the coagulation disorders observed in PLWH with COVID-19 (**Figures 3a, b**). In particular, coagulation factors V (F5) and VIII (F8) were significantly higher in PLWH with COVID-19 than in HCs and PLWH (**Figures 3c, d**). F13A1, which is activated in the final step of the coagulation cascade, induces hemostasis and stabilizes fibrin clots to prevent fibrinolysis (24). We found that F13A1 levels were lower in PLWH with COVID-19 than in HCs, but higher than in PLWH (**Figure 3e**). Additionally, the levels of coagulation factor XIII B chain (F13B) in PLWH with COVID-19 were significantly lower than those in PLWH and HCs (**Figure 3f**). Coagulation factors VII (F7), IX (F9), and XII (F12) were elevated in PLWH with COVID-19 compared with HCs, but no significant differences were observed relative to PLWH (**Figures 3g–i**). VWF is a glycoprotein that binds to F8, protecting it from degradation by vitamin K-dependent protein C (PROC), and triggering platelet aggregation following vascular injury (25). Interestingly, despite the changes in F8 levels, VWF was significantly lower in PLWH with COVID-19 than in PLWH, while PROC did not change significantly. (**Figures 3j, k**).

Our data also showed increased levels of fibrinogen alpha, beta, and gamma chains (FGA, FGB, and FGG) in PLWH with COVID-19 compared to those in HCs (**Figures 3l–n**). These proteins are cleaved into fibrin, which contributes to blood clot formation (26). We detected the dysregulation of multiple serine protease inhibitors in PLWH with COVID-19 (**Figure 3a**). SERPINA5 (**Figure 3o**) is a serine protease inhibitor that inhibits thrombin and is a cofactor for heparin. SERPINA5 is involved in several biological processes, including inflammation, coagulation, and the response to elevated platelet cytoplasmic Ca²^+^ (27). Additionally, we observed dysregulation of various serine protease inhibitors, including SERPINA1, SERPINE2, and SERPINF2. Abnormal angiogenesis resulting from aberrant coagulation has been reported in the lungs of patients with COVID-19 (28). The tissue factor pathway inhibitor (TFPI) is a Kunitz-type serine protease inhibitor that exerts anticoagulant effects by blocking early procoagulant stimuli (29). Our data showed that the TFPI level in PLWH with COVID-19 was higher than that in both the HCs and PLWH (**Figure 3p**). In addition, abnormal angiogenesis resulting from aberrant coagulation led to significant dysregulation of nine angiogenesis-related proteins in the PLWH with COVID-19 group compared to that in the HC group (**Figure 3b**).

These results suggest that, although the two groups of PLWH showed activation of the coagulation and complement cascades, coinfection with SARS-Cov-2 resulted in differences in the expression of associated factors.

### 3.3 Three clusters of proteins relevant to SARS-CoV-2 infection

**Figure 4:**
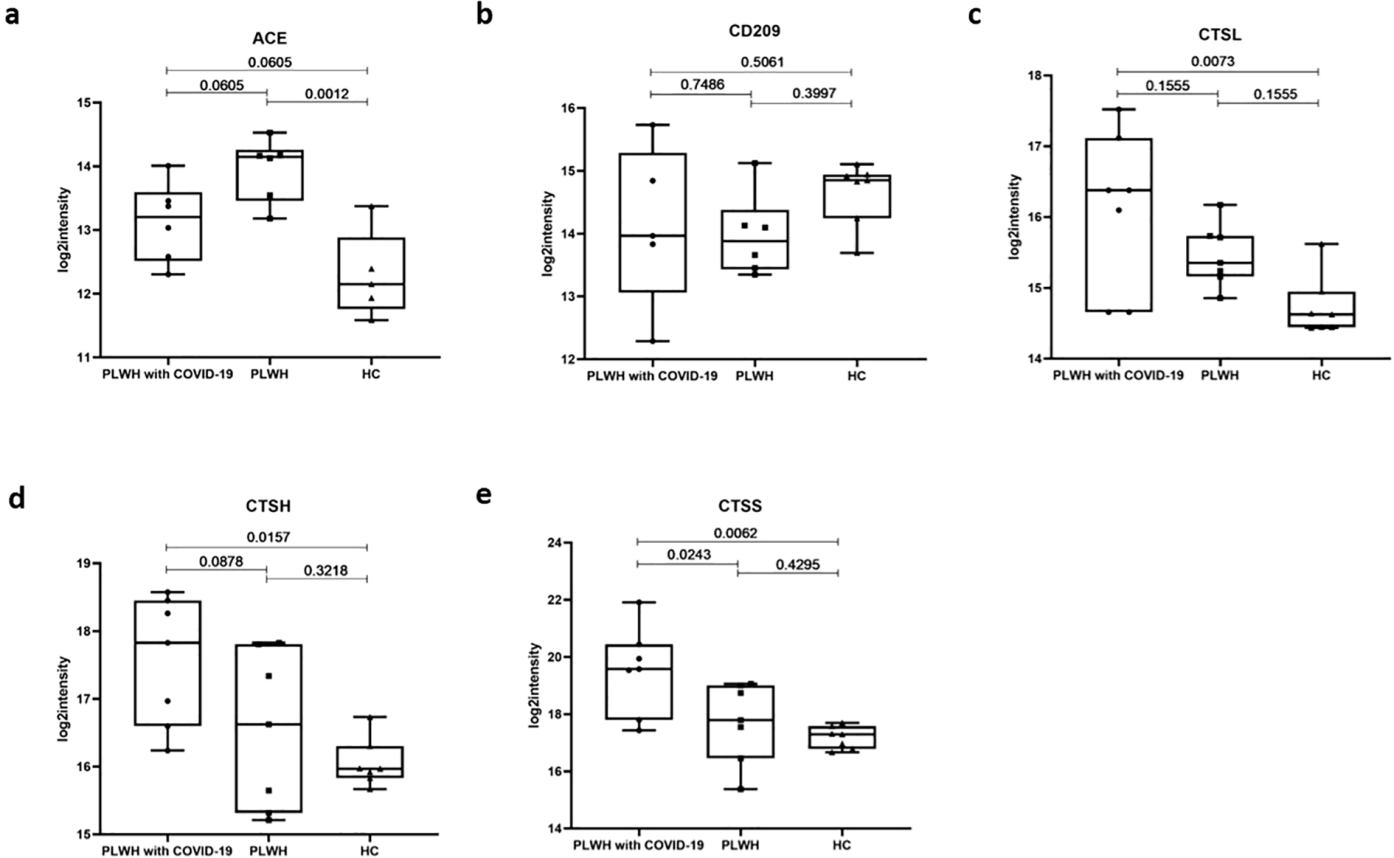
Potential virus receptors and proteases associated with SARS-Cov-2 infection. **(a–e)**. Box plots showing expression levels of ACE **(a)**, CD209 **(b)**, CTSL **(c)**, CTSH **(d)**, and CTSS **(e)** in the PLWH with COVID-19, PLWH, and HCs. Data were normalized, one-way ANOVA and Tukey’s HSD used for statistical analysis.

We focused on several clusters of proteins associated with SARS-CoV-2 infection, including viral receptors, proteases, and transcription factors (TFs). Angiotensin-converting enzyme (ACE) and its homolog ACE2 play crucial roles in the renin-angiotensin system pathway (30, 31). ACE2 was identified as a receptor for the spike (S) protein of SARS-CoV, finally facilitating viral entry into target cells (32). ACE levels were significantly elevated in PLWH relative to HC; however, this difference was not observed in PLWH with COVID-19 (**Figure 4a**). C-type lectin domain family 4 member L (CD209) is expressed on type II alveolar cells in human lungs, which are an important target for SARS-CoV infection, as well as on endothelial cells (33). However, our data showed no significant difference in CD209 levels in the plasma of PLWH with COVID-19 compared with the other two groups (**Figure 4b**). Protease activation of the spike glycoprotein is a critical step for coronavirus entry, and lysosomal cathepsins have been shown to be essential for SARS-CoV and SARS-CoV-2 entry through endocytosis (34). Our results indicated that CTSL, a serine protease involved in the SARS-CoV-2 endosomal pathway, was significantly upregulated in the lungs, spleen, renal medulla, and thyroid (35). In the plasma of PLWH with COVID-19, CTSL levels were higher than those in the HC group, but there was no significant difference compared to the PLWH (**Figure 4c**). However, we found that the levels of other lysosomal proteases, such as CTSH and CTSS, were significantly higher than those the PLWH and HC groups (**Figures 4d, e**). Although further validation is needed, these results raise the possibility that preexisting HIV infection could affect SARS-CoV-2 host cell invasion processes.

**Figure 5:**
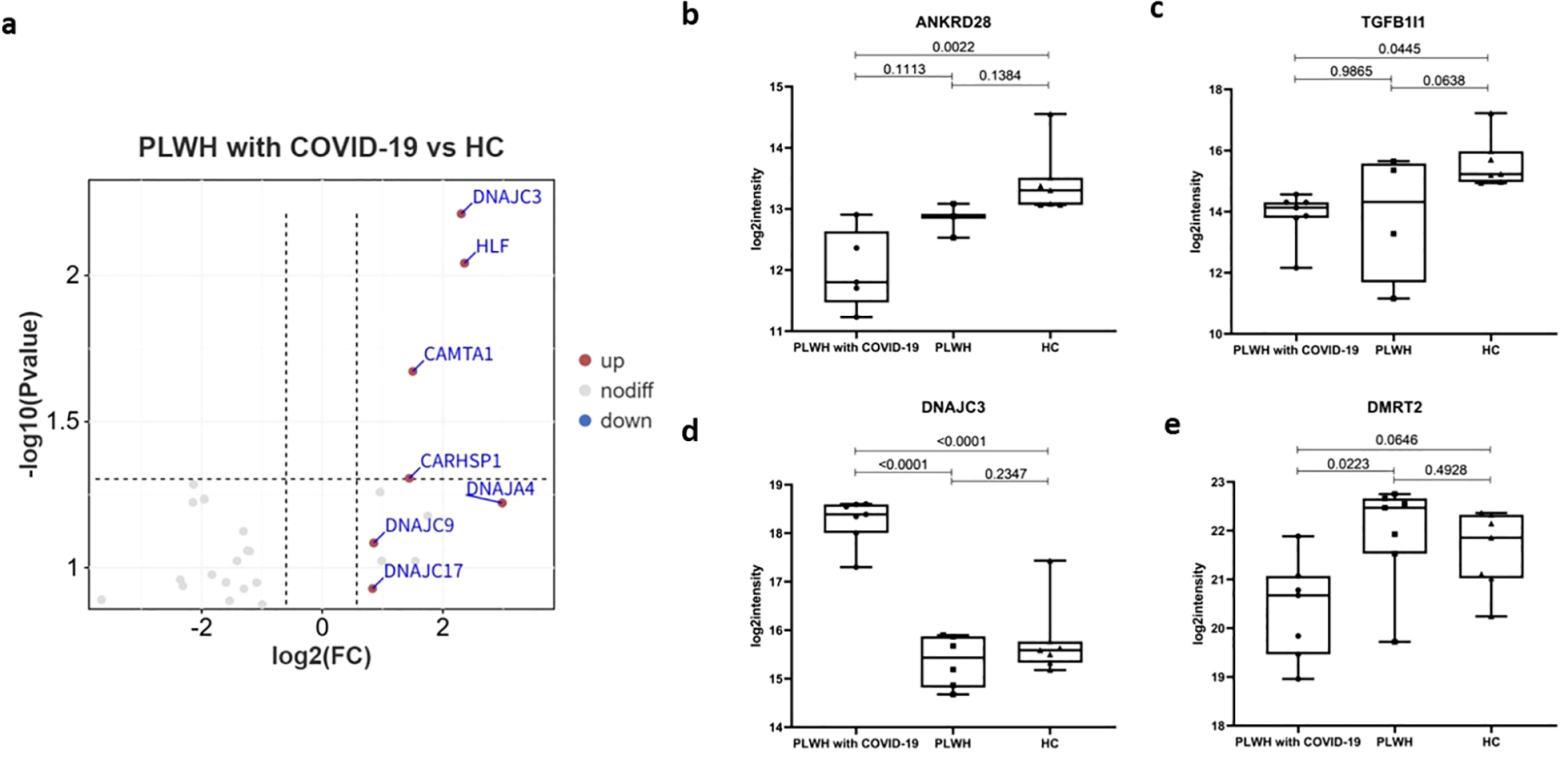
TFs associated with SARS-Cov-2 infection. **(a)** Volcano plot showing the significantly upregulated (red dots, FC > 1.5, two-sided *t* test, *p <*0.05, adjusted by FDR) and downregulated (blue dots, FC < 0.67s, two-sided *t* test, *p <*0.05, adjusted by FDR) TFs between the PLWH with COVID-19 and HCs. b-e. Box plots showing the expression of ANKRD28 **(b)**, TGFβ1I1 **(c)**, DNAJC3 **(d)** and DMRT2 **(e)** in the PLWH with COVID-19, PLWH, and HCs. One-way ANOVA and Tukey’s HSD used for statistical analysis.

After cellular entry, which is mediated by receptors and proteases, SARS-CoV-2 hijacks the host translation machinery and induces a host inflammatory response via TFs, leading to a hyperinflammatory state. This hyperinflammatory state might be associated with clinically observed blood hypercoagulability, as measured by blood tests in patients with COVID-19 (28). Next, we used the Animal Transcription Factor Database for the TF annotation of all DEPs. The DEPs between the two groups, PLWH with COVID-19 and HCs, were annotated to 11 TF families, totaling 27 TFs (**Figure 5a**). Serine/threonine-protein phosphatase 6 regulatory ankyrin repeat subunit A (ANKRD28) is a putative regulatory subunit of protein phosphatase 6 (PP6) that may be involved in the recognition of phosphoprotein substrates. It is involved in PP6-mediated dephosphorylation of NFKBIE, opposing its degradation in response to tumor necrosis factor-alpha. ANKRD28 selectively inhibits the phosphatase activity of PPP1C and targets PPP1C to modulate HNRPK phosphorylation (36, 37). PLWH with COVID-19 had significantly lower plasma ANKRD28 levels than HC but similar levels to PLWH without COVID-19 (**Figure 5b**). KEGG data also revealed enrichment of the NF-κB signaling pathway, but the result was not significant (**Figure 2b**). Members of the MYB family, including DANJ homolog subfamily C member 3 (DNAJC3) was significantly upregulated in the plasma of PLWH with COVID-19 (**Figures 5a, d**). DNAJC3 is involved in the unfolded protein response during endoplasmic reticulum stress. It acts as a co-chaperone of HSPA8/HSC70, stimulating its ATPase activity and potentially inhibiting both the autophosphorylation of EIF2AK2/PKR and its ability to catalyze the phosphorylation of EIF2A (38, 39). Our analysis revealed increased plasma levels of DNAJC9, DNAJC17, and DNAJA4 in PLWH diagnosed with COVID-19, though these elevations were not statistically significant after FDR adjustment. (**Figure 5a**). Transforming growth factor beta-1-induced transcript 1 protein (TGFB1I1) functions as a molecular adapter that coordinates multiple protein-protein interactions at focal adhesion complexes and in the nucleus, regulating the Wnt and TGF-β signaling pathways (40, 41). Although not significantly different from that in the PLWH group, the level of TGFB1I1 in PLWH with COVID-19 was significantly lower than that in the HC group (**Figure 5c**). DMRT2 is a transcriptional activator that directly regulates the early activation of the myogenic determination gene MYF5 by binding to the early epaxial enhancer element in a sequence-specific manner (42, 43). DMRT2 levels in PLWH with COVID-19 were significantly lower than those in PLWH without COVID-19, but showed no significant difference compared to HCs (**Figure 5e**). Patients with COVID-19 and HIV coinfection exhibit unique patterns of TF regulation, and these changes may collectively lead to aberrant regulation of viral replication and enhanced inflammatory responses by affecting signaling pathways such as NF-κB and Wnt.

**Figure 6:**
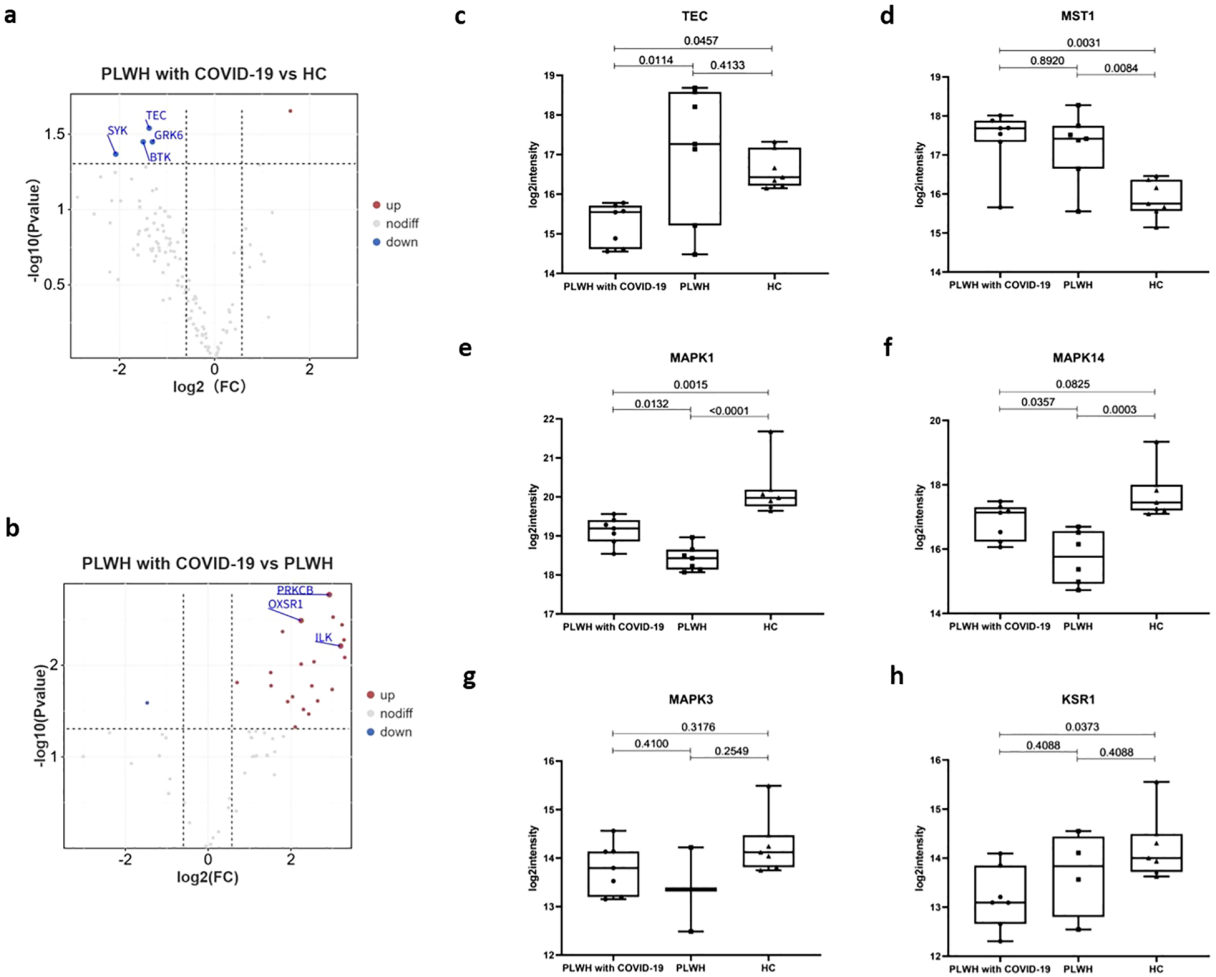
Kinases associated with SARS-Cov-2 infection. **(a)** Volcano plot showing the significantly upregulated (red dots, FC > 1.5, two-sided *t* test, *p <*0.05, adjusted by FDR) and downregulated (blue dots, FC < 0.67, two-sided *t* test, *p <*0.05, adjusted by FDR) kinases between the PLWH with COVID-19 and HCs. **(b)** Volcano plot showing the significantly upregulated and downregulated kinases between the PLWH with COVID-19 and PLWH. c-h. Box plots showing the expression of TEC **(c)**, MST1 **(d)**, MAPK1 **(e)**, MAPK14 **(f)**, MAPK3 **(g)** and KSR1 **(h)** in the PLWH with COVID-19, PLWH, and HCs. One-way ANOVA and Tukey’s HSD used for statistical analysis.

Kinases are a class of enzymes that transfer phosphate groups from high-energy donor molecules, such as ATP, to specific substrates. Protein kinases phosphorylate specific proteins, thereby altering their activity and function. These kinases play a wide range of roles in cell signaling and other complex biological activities. We used the PhosphoSitePlus database to annotate and analyze the kinases. Compared to the HC and PLWH groups, the PLWH with COVID-19 exhibited altered plasma protein levels of multiple kinases (**Figures 6a, b**). Tyrosine protein kinase (TPKs) mediate signal transduction downstream of various transmembrane receptors and regulate several biological processes, including innate and adaptive immunity, cell adhesion, osteoclast maturation, platelet activation, and vascular development (44). Compared to the HC group, several TPKs in the plasma of PLWH with COVID-19 were significantly downregulated, including BTK, SYK, and TEC (**Figure 6a**). TEC is required for TCR-dependent IL2 gene induction and contributes to CD28 signaling (45). The TEC level in PLWH with COVID-19 was also significantly lower than that in PLWH (**Figure 6c**). Macrophage-stimulating 1 (MST1), a novel regulator of cell survival that is closely associated with tyrosine kinase receptor signaling, was significantly upregulated in both PLWH with COVID-19 and PLWH (**Figure 6d**) (46).

Serine/threonine kinases are essential components of the MAP kinase (MAPK) signaling pathway. The MAPK/ERK cascade mediates diverse biological functions, such as cell growth, adhesion, survival, and differentiation, through the regulation of transcription, translation, and cytoskeletal rearrangements (47, 48). MAPK1/ERK2 and MAPK3/ERK1 are two key MAPKs that play crucial roles in the MAPK/ERK cascade (49). In PLWH with COVID-19, MAPK1 levels were intermediate between PLWH and HC group, whereas MAPK14 was elevated relative to PLWH but not significantly different from HCs. (**Figures 6e, f**). In contrast, MAPK3 levels did not exhibit significant changes (**Figure 6g**). Kinase Suppressor of Ras 1 (KSR1) is part of a multiprotein signaling complex that promotes the phosphorylation of Raf family members and activates downstream MAPKs (50). KSR1 levels in PLWH with COVID-19 were significantly lower than those in the HC group (**Figure 6h**). This finding is consistent with the significant enrichment of the MAPK signaling pathway observed in our proteomic data (**Figures 2a, b**). Together, these changes may contribute to the pathophysiological features of HIV and COVID-19 coinfection through mechanisms that affect the immune response and cellular signaling.

## 4 Discussion

In recent years, a growing number of studies have demonstrated the possible connections between various diseases. Therefore, the interactions among different diseases represent a highly promising field that warrants further investigation (51). The global impact of COVID-19 has been considerable, presenting major challenges to public health, food systems, and the workforce. HIV, a member of the *Retroviridae* family within the genus *Lentivirus*, has a wide-ranging impact on individual health, households, communities, and national economic and social well-being (52). While PLWH represent a heterogeneous group, those with advanced or untreated HIV may experience poorer outcomes when coinfected with COVID-19. However, the molecular mechanisms underlying the poor COVID-19 prognosis in PLWH remain unclear. In this study, we report the proteomic profiles of hospitalized patients with HIV combined with SARS-CoV-2 infection. Our results demonstrate that multiple biological and pathological processes are regulated in PLWH with COVID-19, including systemic damage, dysregulation of the complement and coagulation cascades, a pronounced proinflammatory response, and upregulation of viral entry factors.

Disease severity is primarily correlated with inflammatory mediators and networks, including acute-phase response proteins, leukocyte-mediated immunity, and neutrophil degranulation. Several proteins linked to the acute phase response, such as CRP, SERPINA3, SAA1 and SAA2, have been shown to increase in the plasma of patients with severe COVID-19 using MS-based data-independent analysis (53). We observed a similarly elevated acute-phase response in the plasma of PLWH with COVID-19 (**Figures 1c–e**). Proteomic profiling has also identified numerous host proteins associated with HIV infection, including CD14, CD44R5, vinculin, and S100A9 (54). In our study, we observed a significant upregulation of S100A9 and its heterodimer partner S100A8 in PLWH with COVID-19 compared to HCs (**Figure 1c**). Additionally, we noted the upregulation of a panel of angiogenesis-related markers in these patients. Several key factors in the coagulation pathway, including F5, F12, and SERPINA5, were also elevated in PLWH with COVID-19. Dysregulation of the coagulation cascade is consistent with the proteomic findings in patients with COVID-19 with critical disease or postmortem observations (35, 55). Chronic inflammation due to HIV-induced immune system dysregulation can lead to endothelial cell dysfunction and platelet activation, which together increase the risk of arterial thrombosis (56). VTN is a 75 kDa multifunctional glycoprotein, also known as the S protein of the complement system. Predominantly produced in the liver, VTN plays a crucial role in tissue remodeling by binding to various integrins through its Arg-Gly-Asp motif and regulating cell adhesion. It also regulates blood system-related protease cascades, such as coagulation and fibrinolysis, by interacting with heparin and thrombin-antithrombin III complexes (57). We observed a significant upregulation of VTN in the plasma of PLWH with COVID-19. A recent study highlighted that VTN, along with other proteins involved in extracellular matrix organization, belongs to a cluster of molecules that are significantly upregulated only in patients with COVID-19 with fatal pneumonia compared to those with severe pneumonia requiring intensive care unit (ICU) admission and those with pneumonia not requiring ICU admission (58). Our data showed that, although the two groups of PLWH showed activation of the coagulation and complement cascades, coinfection with SARS-Cov-2 resulted in differences in the expression of associated factors. HIV and SARS-CoV-2 coinfection may exacerbate coagulation dysregulation through multiple mechanisms, increasing the risk of thrombotic events and disease severity. In-depth research on interactions within the coagulation cascade is crucial for optimizing clinical management and improving patient outcomes.

As indicated by previous studies, a lower CD4 count in PLWH is strongly correlated with an increased chance of SARS-CoV-2 positivity (59). These data highlight the urgent need for mechanistic studies to better illustrate how HIV-associated immunocompromise influences the acquisition and clearance of infections. In this study, we conducted GO and KEGG analyses to examine the association between COVID-19 and HIV. DEPs were highly enriched in processes involved in the complement and coagulation cascades. Additionally, we found that several DEPs are involved in signaling pathways, such as MAPK and NF-κB signaling pathways. MAPK signaling pathway plays a crucial role in signaling cascade responses to various cellular stimuli, including viral infections. Activation of the MAPK/NF-κB signaling pathway following HIV infection leads to the sustained release of inflammatory cytokines, which not only supports viral replication but may also contribute to the development of comorbidities (60, 61). The SARS-CoV-2 S1 spike protein can alter MAPK signaling pathways and promote COVID-19-characteristic cytokine production in relevant human lung epithelial and intestinal epithelial cells, which may contribute to COVID-19 cytokine storm pathology (62). Further studies are needed to determine the role of the MAPK signaling pathway in HIV and SARS-Cov2 coinfection.

Our study has some limitations. Owing to the limited sample size, we were unable to include patients with COVID-19 without HIV infection as a control group, and most HIV-positive inpatients had complicated comorbidities. Another limitation is the age disparity between the HC group and the two PLWH groups, as the HC group was significantly younger. Since age influences immune and metabolic protein expression, this imbalance may introduce bias in our analysis. Future studies should employ age-matched cohorts to validate our findings and minimize age-related confounding effects. Additionally, the relatively small number of patients limits our analysis, and the investigation of comorbidities requires further studies in larger independent cohorts. Furthermore, owing to these limitations, we could not analyze the COVID-19 severity in patients with HIV coinfection.

In summary, we quantified 5,373 proteins across 21 samples and identified significant alterations in PLWH with COVID-19 compared to HC, with 209 DEPs. Compared with PLWH, PLWH with COVID-19 exhibited 568 DEPs. This proteomic atlas uncovered potential molecular targets, signaling pathways, small molecule compounds, and promising biomarkers for HIV and SARS-Cov-2 coinfection. These findings may contribute to a more precise diagnosis and treatment of patients with HIV and COVID-19.

## Data Availability

The original contributions presented in the study are included in the article/**Supplementary Material**. Further inquiries can be directed to the corresponding authors.
